# Annual Meteorological Table for Derby

**Published:** 1813-04

**Authors:** 


					?.92 Mr. Swamvick's Meteorological Tablefor Derby.
ANNUAL METEOROLOGICAL TABLE >> DERBY.
Lah 52? 58'N. Ion. 1? 30' IV.
"By Mft. SwAKwrcK.
1812.
THERMOMETER, KORTH EXPOSURE.
January
February
March
April
May
June
July
i A ugitst
Scptem.
October
Novem.
! Decern,
fl)
19
20
SO
15
22
7
9
18
21
4
1
1
47?
52
58
55
70
72
78
72.
64
59
49
44
W
s
sw
SE
SE
E
SE
S
SW
s
s
SE
?-J
25?
SO
,23
26
31
84
35
38
30
27
23
19
NW
SW
SE
N
W
NW
S
E
S
NW
E
NW
^ Si
42?
45
44
49
59
CO
60
61
58
49
40
34 I
31?
35
32
33
41
42
47
47
42
37
32
29
-J
ka-
36?
40
38
41
50
51
53
54
50
43
36
32
12?
10
14
13
18
14
14
9
16
15
9
9
BAROMETER.
f 170 rent above the 1
\ Level of the Sea. j
WEATHER
?
30.18
30.03
30.31
30.00
30.11
30.31
30.35
30.15
30.20
29.80
30.33
30.48
N
S
N
NW
S
NE
SE
S
SW
S
NE
NE
29.12
29.15
29.13
29.30
29.40
29.23
29.S6
29.75
29.55
28.34
29 21
29.05
S
S
S
W
W
W
W
NE
N
S
NE
NE
5*
ts ?
CT*
29.82
29.59
29.72
29.76
29.73
29.83
29.98
29.93
29.69
29.26
29.77
29.86
.50
? 61
? 54
? 31
? 27
.39
.35
.29
.37
.51
.-19
.39
25
20
16
20
18
19
14
19
22
13
19
21
6
9
15
10
13
11
16
12
8
18
U
10
a
2
=8
2
4
2
12
8
8
4
6
16
1
9| 7
131 9l
Thermometer.
Highest . . . July
Lowest . . . Dec.
Greatest 7 ,T
Variation J
Mean for the Day .
Mean for the Night .
Annual Mean . . .
78?
19
18
50
37
43
Wind.
SE
NW
ANNUAL RESULT
Barometer.
Highest . December 7, 3048
Lowest . October 19, 28.34
Greatest)
Range ( FebrUar* -65
Annual Mean . . . 29.74
S.
Wind.
NE
S
Quantity of
Rain for
the Year.
Inches 23.79
Wind. Times.
N&NE
E & SE
S & SW
W&NW
85
56
119
106
366
Weather.Days.
Fair . . 227
Wet . .139
366

				

